# Low CT temporal sampling rates result in a substantial underestimation of myocardial blood flow measurements

**DOI:** 10.1007/s10554-018-1451-9

**Published:** 2018-10-04

**Authors:** Marly van Assen, Gert Jan Pelgrim, Emmy Slager, Sjoerd van Tuijl, U. Joseph Schoepf, Rozemarijn Vliegenthart, Matthijs Oudkerk

**Affiliations:** 1 Department of Radiology, Center for Medical Imaging-North East Netherlands, University of Groningen, University Medical Center Groningen, Groningen, The Netherlands; 2 LifeTec Group BV, Eindhoven, The Netherlands; 3 Medical University South Carolina, Charleston, SC USA; 4 Center for Medical Imaging-North East Netherlands, University of Groningen, University Medical Center Groningen, EB44, Hanzeplein 1, 9713 GZ Groningen, The Netherlands

**Keywords:** Perfusion imaging, Computed tomography, Cardiac imaging techniques

## Abstract

The purpose of this study was to evaluate the effect of temporal sampling rate in dynamic CT myocardial perfusion imaging (CTMPI) on myocardial blood flow (MBF). Dynamic perfusion CT underestimates myocardial blood flow compared to PET and SPECT values. For accurate quantitative analysis of myocardial perfusion with dynamic perfusion CT a stable calibrated HU measurement of MBF is essential. Three porcine hearts were perfused using an ex-vivo Langendorff model. Hemodynamic parameters were monitored. Dynamic CTMPI was performed using third generation dual source CT at 70 kVp and 230–350 mAs/rot in electrocardiography(ECG)-triggered shuttle-mode (sampling rate, 1 acquisition every 2–3 s; z-range, 10.2 cm), ECG-triggered non-shuttle mode (fixed table position) with stationary tube rotation (1 acquisition every 0.5–1 s, 5.8 cm), and non-ECG-triggered continuous mode (1 acquisition every 0.06 s, 5.8 cm). Stenosis was created in the circumflex artery, inducing different fractional flow reserve values. Volume perfusion CT Myocardium software was used to analyze ECG-triggered scans. For the non-ECG triggered scans MASS research version was used combined with an in-house Matlab script. MBF (mL/g/min) was calculated for non-ischemic segments. True MBF was calculated using input flow and heart weight. Significant differences in MBF between shuttle, non-shuttle and continuous mode were found, with median MBF of 0.87 [interquartile range 0.72–1.00], 1.20 (1.07–1.30) and 1.65 (1.40–1.88), respectively. The median MBF in shuttle mode was 56% lower than the true MBF. In non-shuttle and continuous mode, the underestimation was 41% and 18%. Limited temporal sampling rate in standard dynamic CTMPI techniques contributes to substantial underestimation of true MBF.

## Introduction

Coronary artery disease (CAD) is one of the major causes of death in industrialized countries, and one of the leading causes of years of life lost due to premature mortality [1, 2]. Multiple myocardial perfusion imaging (MPI) modalities can be used to evaluate the hemodynamic significance of CAD non-invasively [3–5]. Recent technical developments in the field of computed tomography (CT) make this imaging modality a suitable candidate for cardiac perfusion measurements [6]. Dynamic CTMPI has several advantages over competing imaging modalities, namely cost, availability, spatial resolution and the linear relation between Hounsfield unit (HU) enhancement and contrast in the myocardium, facilitating quantification. Using a combination of CT angiography (CTA) and CTMPI the morphological and functional aspects of CAD can be evaluated with a single non-invasive imaging modality.

In a clinical setting, MPI evaluation is mostly based on visual assessment of differences in regional enhancement by trained experts [7]. Qualitative analysis relies on the assumption that there is normally perfused myocardium present as remote area. This assumption is not needed for quantitative evaluation [8, 9]. Therefore, quantitative analysis offers several advantages, including the potential to accurately grade the severity of ischemia and the ability to detect global ischemia and multi-vessel disease [8].

Dynamic CTMPI has been found to underestimate myocardial blood flow (MBF) compared to positron emission tomography, the current gold standard for in-vivo quantification of MBF. Studies with positron emission tomography report stress MBF values ranging between 3 and 5 mL/min/g [10, 11], whereas previous studies using dynamic CTMPI report stress MBF values of 1.0–1.4 mL/min/g [12–15]. Ishida et al. report an underestimation of 23–41% of CT-measured MBF values compared to true values in a simulation study evaluating the effects of temporal sampling [16].

A stable calibrated HU-measurement during the inflow of contrast is essential for accurate quantitative analysis of myocardial perfusion in CTMPI. Recent studies of Bindschadler et al. and Ishida et al. suggest that the underestimation of dynamic CTMPI-determined MBF is partially caused by the limited temporal sampling rate of current dynamic CTMPI modalities [16, 17].

In this proof of principle study we evaluate the effect of increased temporal sampling rates on quantification of MBF in dynamic CTMPI with 3rd generation dual-source CT in an ex-vivo porcine heart model.

## Materials and methods

Three hearts were obtained from Dutch landrace hybrid slaughterhouse pigs. All protocols were in accordance with the EC regulation 1069/2009 regarding the use of slaughterhouse animal material for diagnosis and research, supervised by the Dutch Government (Dutch Ministry of Agriculture, Nature and Food Quality), and approved by the legal authorities of animal welfare (Food and Consumer Product Safety Authority). The transport of the hearts from the slaughterhouse to the CT-scanner took approximately 3–4 h, during which the physiological preservation was optimal.

### Experimental setup

The three porcine hearts were perfused using an isolated heart model (Physioheart©, LifeTec Group, Eindhoven, The Netherlands) in Langendorff mode [18, 19]. A more detailed description of the model application in CT has been published [19, 20].

Blood was obtained from slaughterhouse pigs and pumped from a venous reservoir into the coronaries by means of retrograde flow through the aorta, using a centrifugal pump (BioMedicus, Medtronic, Minneapolis, MN, USA). The aortic valve was closed due to the retrograde pressure on the valve, ensuring that all circulating blood passed through the coronary arteries. The blood was oxygenated with a gas mixture (20% O_2_, 75% N_2_, and 5% CO_2_) at a temperature of 38 °C using an oxygenator-heat exchanger (AFFINITY^®^ NT Oxygenator; Medtronic). Blood glucose levels were kept at constant level between 5 and 7 mmol/L by adding a mixture of glucose and insulin.

The porcine hearts began to contract spontaneously after the perfusion through the myocardium was restored. Defibrillations (10–30 J) and an external pacemaker (Model 5375, Medtronic) were used to acquire a stable sinus rhythm.

A stenosis was created with an adjustable inflatable cuff around the proximal circumflex artery (Cx). The cuff allowed control of the degree of stenosis. Furthermore, a pressure wire was inserted in the Cx to monitor the pressure drop across the stenosis and to measure fractional flow reserve (FFR). Adjustment of the cuff was used to create multiple FFR based stenosis grades: FFR 1.00–0.90, 0.80, 0.70, 0.60, 0.50 and total occlusion. The hearts were first scanned without any stenosis, subsequently followed by increasing FFR stenosis grades.

### CT protocol

A third generation dual source CT system (Force, Siemens Healthineers, Forchheim, Germany) was used to perform dynamic CTMPI.

Frontal and lateral scout images were used to determine the region of interest for the perfusion scans. To confirm the field of view and assess background-noise and artefacts, a baseline non-contrast scan was performed at 70 kV and 20 mAs for each heart. Subsequently, at each FFR value, a dynamic CTMPI scan was performed. Dynamic CTMPI was performed using three different scan modes.

The first scan mode, a conventional electrocardiography (ECG)-triggered shuttle mode was used with a tube voltage of 70 kV, tube current time product 350 mAs per rotation, and gantry rotation time of 250 ms. The shuttle mode uses alternating table positions, with the table moving back and forth to scan the entire heart. The shuttle mode scans were performed during end-systole. These acquisition parameters resulted in a z-range of 10.2 cm, covering the whole heart. A scan was made every other heartbeat, resulting in one image every 2–3 s on average.

A second scan mode, an ECG-triggered non-shuttle mode, was used with a fixed table position, resulting in a z-range of 5.8 cm and an acquired image approximately every half second. Other acquisition parameters were: tube voltage 70 kV, tube current time product 230 mAs per rotation, and a gantry rotation time of 250 ms. Scans were performed during end-systole. Due to the smaller z-range the non-shuttle dynamic scan mode scans were acquired at the mid-ventricular level, covering the whole heart. A scan was made every heartbeat, resulting in one acquisition per 0.5–1 s. This scan protocol was designed for research purposes only and is not used in clinical practice.

The third scan mode was a continuous mode (continuous acquisition), again with a fixed table position, resulting in a 5.8 cm z-range, comparable to the method used in a previously published study [21]. The continuous scans were not ECG-synchronized and 16 images were acquired every second. Other acquisition parameters were: tube voltage 70 kV, tube current time product 230 mAs per rotation, and a gantry rotation time of 250 ms. The continuous scans were acquired at the mid-ventricular level using a single source. As with the non-shuttle mode, this scan protocol was designed for research purposes only and should not be used in clinical practice.

For all dynamic CTMPI scans, 15 mL of ioxaglate (Hexabrix, 320 mg/mL, Guerbet, Paris, France) contrast agent was injected with an injection rate of 3 mL/s. The dynamic scans started 5 s prior to injection of contrast agent. During initial inflow and outflow of iodine contrast through the myocardium, data were collected over a scan-time of 60 s. To allow myocardial enhancement to return to baseline values there was a 5-min interval between each scan. To prevent the build-up of contrast agent and to prevent baseline shifts, the total blood reservoir was large, 20 L.

A part of the inflow tube of the perfusion system was looped through the field of view of the perfusion scans, acting as a surrogate for the human aortic blood flow of iodine contrast. From this substitute aorta an AIF was derived, which was used for the quantification of MBF.

### MBF calculations

True MBF is defined as the flow in ml per gram of myocardial tissue per minute in a non-stenotic state. Each heart was weighed after the experiments. The true MBF was calculated according to the weight (g) of the heart and the input flow (mL/min) during FFR grade 1.0–0.9.

$$MB{F_{true}}=\frac{{Input~flow}}{{Heart~weight}}\quad {\text{for weight based measurements}}$$

Volume perfusion CT (VPCT) myocardium software (MMWP VA41A, Siemens) was used to analyze the ECG-triggered shuttle and non-shuttle scans. For the continuous scans, MASS research version software (Medis, Leiden, The Netherlands) was used to extract the arterial input function (AIF) and tissue attenuation curve (TAC) per segment. An in-house Matlab script (The MathWorks Inc., Natick, MA) was used to calculate the MBF using the same mathematical perfusion model as the VPCT software (upslope method). VPCT software calculates MBF according to the following equation:

$$MBF={\text{MaximumSlope}}\;({\text{TAC}})/{\text{MaximumValue}}\;({\text{AIF}})$$

Where the *MaximumSlope* (*TAC*) is the maximum upslope of the TAC curve of the myocardium and the *MaximumValue* (*AIF*) is the peak value of the AIF curve measured in the aorta.

Myocardial segments were manually defined. The American Heart Association 16-segment model was used to classify the myocardial segments at basal, mid-ventricular and apical levels [22]. Based on the total occlusion scans, segments perfused by the Cx-artery were considered ischemic, all other segments were considered non-ischemic. Mean values of MBF (mL/g/min) were calculated per segment [22].

For the ECG-triggered non-shuttle mode, VPCT-calculated MBF and Matlab-calculated MBF were compared for the non-ischemic segments of all hearts at non-occluded state. They showed a high correlation with a correlation coefficient of 0.89. Analysis with VPCT software resulted in medians of 1.22 [interquartile range (IQR) 1.12–1311.31] and analysis using Matlab resulted a median of 1.14 (IQR 1.02–122). No significant differences were found between the MBF values calculated with VPCT and with Matlab (p = 0.053 Mann–Whitney U test).

### Statistical analysis

Statistical analysis was performed using SPSS 23 (IBM Corp, Armonk, NY, USA). A mixed linear model was used to compare the MBF values between the different scan modes, corrected for repeated measures and between-heart variability. Pearson’s correlation coefficient was used to evaluate the correlation between input flow and MBF.

## Results

Heart rate (range 107–115 bpm), model blood flow (range 1.0–1.2 L/min) and blood pressure (range 73–84 mmHg) were generally stable for all three hearts during the experiments.

For each scan mode 11 non-ischemic segments per stenosis grade were analyzed (55 segments for all perfusion grades) resulting in 165 analyzed segments in total.

True global MBF as measured by the artificial pump circulation and the actual myocardial muscle weight, was calculated to be a median of 1.78 (1.77–1.79) mL/g/min for heart 1, 2.80 (2.72–2.80) mL/g/min for heart 2, and 1.88 (1.80–1.91) mL/g/min for heart 3, averaged over the three scan modes in non-occlusion state.

### Comparing MBF for temporal sampling rates

**Fig. 1:**
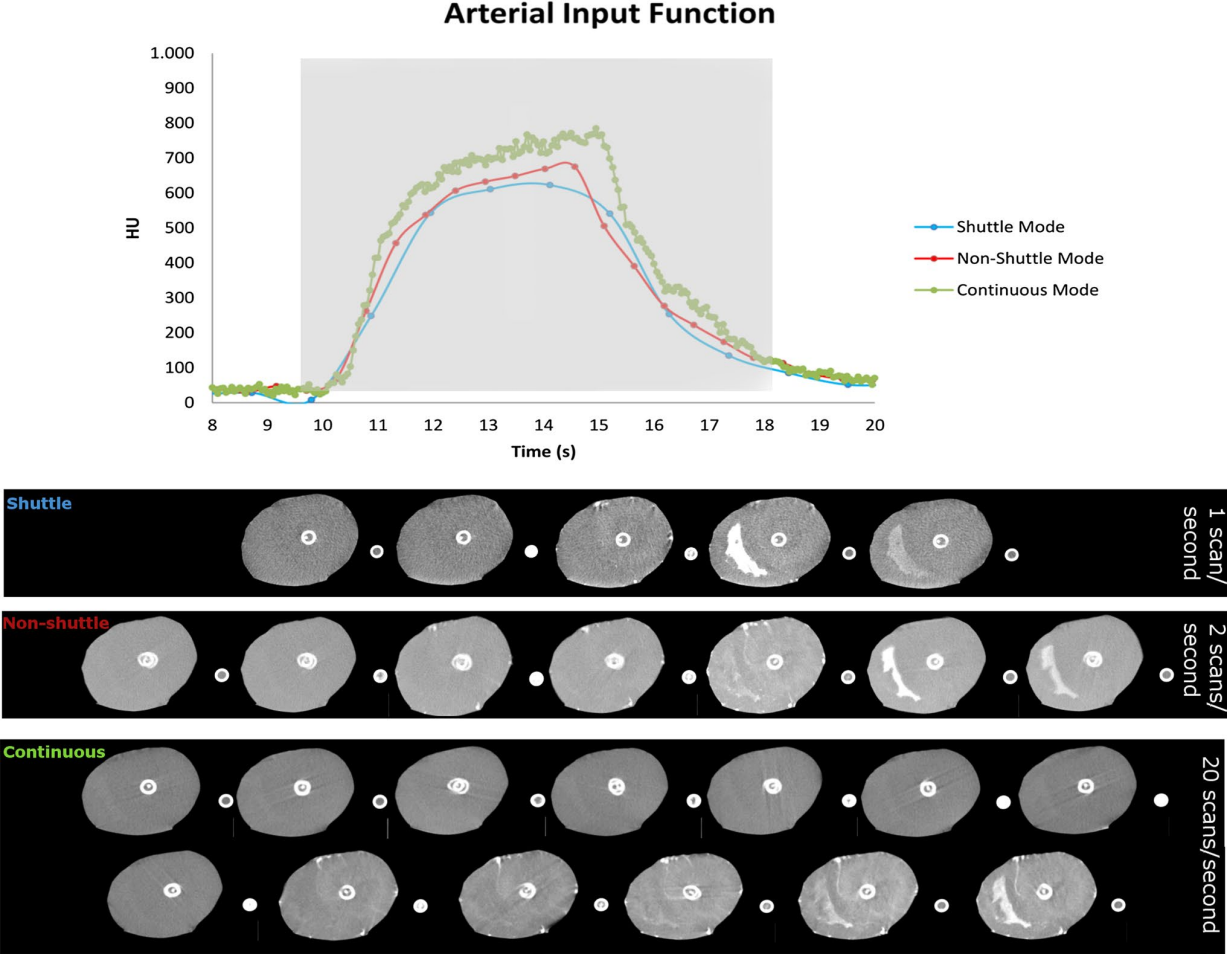
The arterial input functions (AIF) of shuttle (blue), non-shuttle (red) and continuous (green) perfusion mode with correlating CT images (Light grey square)

The arterial input functions between shuttle, non-shuttle and continuous mode are illustrated in Fig. 1, with correlating CT images. Compared with the shuttle mode, there are double data-points in the AIF using the non-shuttle mode and 30 times more data-points using the continuous mode. The AIF of the non-shuttle and continuous mode show the same shape, whereas the AIF in shuttle mode shows a different shape.

**Fig. 2:**
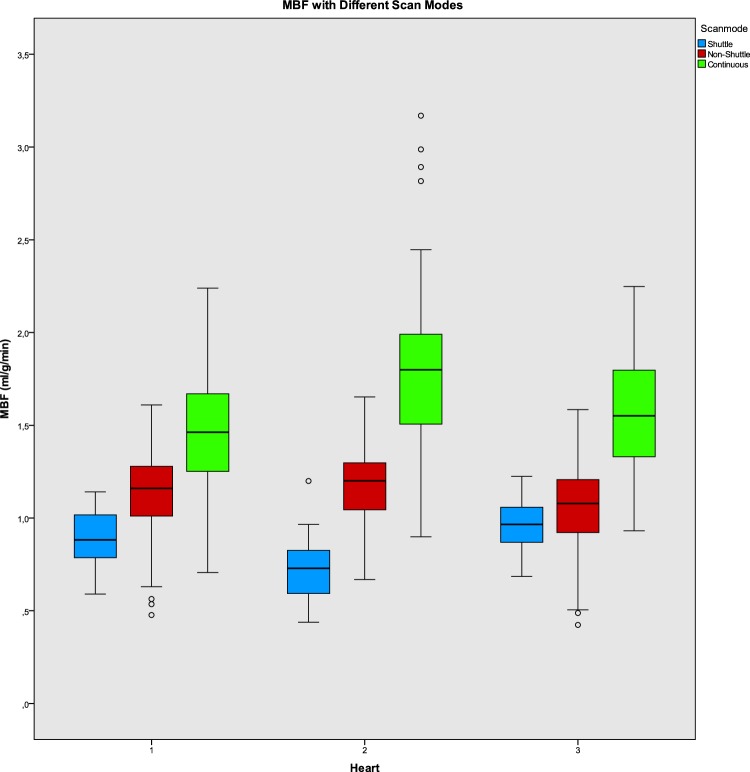
The median myocardial blood flow (MBF) in mL/min/g and interquartile range (IQR) for non-ischemic segments per heart for shuttle, non-shuttle and continuous CT perfusion scan mode

Figure 2 shows the median MBF values and IQR per heart for the three different scan modes with significant differences between each scan mode. The MBF values increased with increasing temporal sampling rate. The mixed linear model found significant differences in MBF between shuttle, non-shuttle-mode and continuous mode for all stenosis grades (p-values < 0.001 for all stenosis grades), with a median MBF of 0.87 [interquartile range (IQR) 0.72–1.00], 1.20 (1.07–1.30) and 1.65 (1.40–1.88) for shuttle, non-shuttle and continuous mode respectively. The continuous mode provided the highest absolute MBF values.

**Fig. 3:**
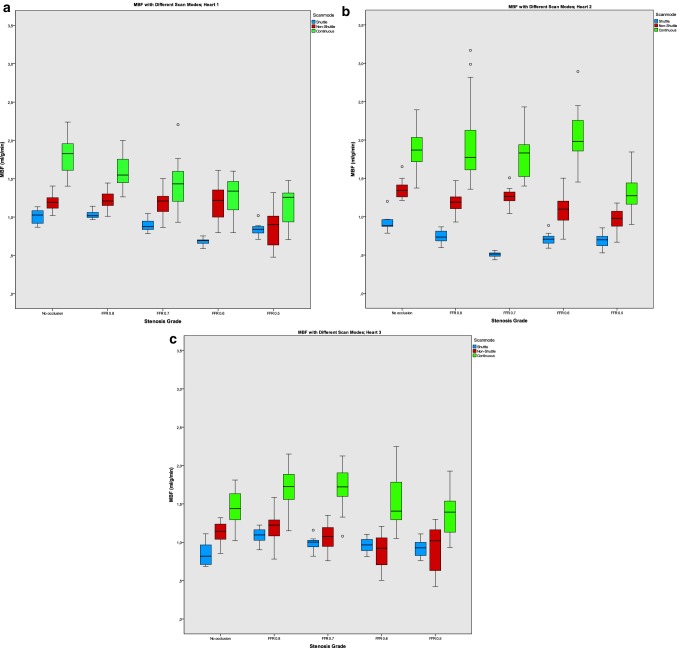
The median myocardial blood flow (MBF) in mL/min/g and interquartile range (IQR) for the different stenosis grades per scan mode for non-ischemic segments and the input flow in mL/min for heart 1 (**a**), heart 2 (**b**) and heart 3 (**c**)

Figure 3a–c shows the median MBF and IQR for the different stenosis grades per scan mode for the non-ischemic segments of each heart. With increasing stenosis grade the input flow decreased. The MBF showed a decrease with increasing stenosis grades, corresponding with the decrease in input flow. In non-ischemic segments, the Pearson’s correlation coefficient (r) (r = 0.629, 0.503 and 0.681) showed a strong correlation between the decrease in input flow and in MBF using shuttle, non-shuttle and continuous mode respectively.

### Absolute MBF values

Compared to the true median MBF, the median MBF (averaged over all hearts at FFR 0.90) in non-ischemic segments was 56% lower for shuttle mode, 41% lower for non-shuttle mode and 18% lower for continuous mode. Increasing the temporal sampling rate resulted in a MBF increase of 44–98% compared to the clinically used shuttle mode. The percentual increase in MBF values compared to the true MBF was significant between shuttle to non-shuttle mode (p-value < 0.1001) and between non-shuttle and continuous mode (p-value < 0.1001).

## Discussion

This study analyzed the influence of different temporal sampling rates on the quantification of MBF in an ex-vivo porcine heart model. We report several important findings. First, this study shows that an increase in temporal sampling rate affects the calculated MBF values. The clinically used shuttle mode resulted in a median MBF of 0.87 (0.72–1.00) mL/g/min whereas non-shuttle and continuous mode provided significantly higher median MBF values of 1.20 (1.07–1.30) and 1.65 (1.40–1.88). Secondly, the continuous scan mode provided more accurate MBF values compared to the true MBF values than shuttle and non-shuttle mode. In shuttle mode the median MBF was 56% lower than the true value, compared with 41 and 18% for non-shuttle and continuous mode, this is a significant reduction in underestimation for the non-shuttle and continuous mode.

The temporal sampling rate has a large influence on the shape of the AIF curve in shuttle mode caused by the timing of the limited acquisitions. Values from those acquisitions determine the peak of the AIF, whereas with higher temporal sampling rates, the shape of the curves becomes less dependent on timing.

The variability in MBF registration increased with increasing temporal sampling rates. This could reflect the higher accuracy of the curve fitted through the points. With an increased number of measurements, the curve fitting will be more accurate. The continuous signal registration is an advantage in the detection of small changes in MBF. These small changes are missed in scan modes with low temporal sampling rates but will show improved registration in scan modes with higher temporal sampling rates.

The decreasing trend in MBF values with increasing stenosis grades could be partially explained by the decrease in input flow (see Fig. 3a–c) at higher stenosis grades. Higher stenosis grades caused an increase in pre-stenotic pressure in the system. As a result of this increased pressure the model automatically decreased the input flow to maintain constant pressures in the heart and coronaries. The decrease in input flow was present in stenosis grades from an FFR grade of 0.7. Another factor is the influence of the stenosis on presumed non-ischemic segments and deterioration of the heart over time. The fact that the overall status of the heart declined over time could result in a decrease in MBF in the non-shuttle scans, which were taken last. The difference between shuttle, non-shuttle and continuous mode-measured MBF could therefore be underestimated. However, the maximum time between the first and the last scan mode was kept to 10 min to minimize these effects.

The concept of MBF quantification was described decades ago for cine CT. Dynamic scan modes were performed on electron beam tomography scanners [23] and their preceding technology, the “dynamic spatial reconstructor” [24]. In-vivo animal studies showed the capability of these dynamic approaches to provide accurate quantitative assessment of regional MBF using temporal sampling comparable to the non-shuttle dynamic scan mode on dual-source CT [26–27]. A study in eight dogs by Weiss et al. showed excellent correlation with an estimate slope of 0.99 for the correlation between electron beam tomography-determined MBF and microsphere-determined MBF for a broad flow range, from 0.04 to 5.9 mL/g/min [26]. Although these results show a high correlation, the baseline MBF measured by microspheres [190 (12) mL/min/100 g] was higher than the MBF measured with cine CT perfusion [148 (14) mL/min/100 g]. It should be noted that microspheres reflect the MBF distribution but not the absolute coronary flow.

In our study, the values for MBF increased with the use of a higher temporal sampling, however, the MBF value is still underestimated. The reason for this underestimation could be that VPCT is calculating the K1-Patlak equivalent instead of MBF, as described by Ishida et al. [16]. The Patlak model is a tracer kinetic model, which describes the transfer constant (K1) of contrast from the blood to the myocardium [28]. In order to calculate the MBF the following equation is then used [15, 29]:

$$F=K1/E$$

where *E* is the extraction fraction (dimensionless) and *F* is the MBF (mL/g/min).

Ishida et al. showed that K1, calculated with Patlak and the VPCT software showed a linear relationship, however, when the K1 VPCT values were transformed to actual flow values according to the equation above, they showed more accurate MBF values compared to their simulated true MBF [16]. A variety of tracer kinetic models, with varying complexity, has been used to calculate MBF in magnetic resonance imaging and CT studies [28]. Other tracer kinetic models, more suitable to model the dynamics of the contrast agent, could give more accurate estimates of MBF. Since the underestimation is less with higher temporal sampling rates than with low temporal sampling rates, better models will only solve this problem partially.

Validation studies on dynamic CTMPI using animal models are scarce, however there are validation studies using MRI perfusion in animal models. Schuster et al. developed and validated an isolated pig heart model for evaluation of cardiovascular magnetic resonance imaging techniques [30]. Another study by Schuster et al. showed that an isolated pig heart provides greater control and reproducibility when compared to an in-vivo animal heart model [31].

During the experiment the input flow was kept constant for each heart. Due to the difference in heart sizes, the MBF (mL/g/min) varied between the hearts, since larger hearts need a higher input flow in order to reach the same pressure and perfusion flow. Therefore, it was decided to calculate the true MBF separately for the three hearts and for different scan modes. Since the input flow varied minimally between the three different scan modes for one heart, the MBF was averaged over the three scan modes.

This study has several limitations. Only a small number of porcine hearts were used. However, MBF was calculated for multiple segments for each heart, resulting in the evaluation of nearly 165 segments per scan mode. The segments were defined manually, which can cause differences between scans. However, automatic segmentation software was not possible due to the experimental set-up, because the automatic software requires a contrast-filled left ventricle. The segments were drawn strictly according to AHA-segmentation guidelines to minimize variations.

The retrograde flow of blood is different from in-vivo experiments. The flow passes the aorta in a retrograde fashion before entering the coronary arteries and the inflow tube serves as an aortic substitute, whereas in in-vivo experiments, the blood flows from the left ventricle to the coronary arteries. As a consequence, the whole blood and contrast volume, used for deriving the AIF curve, passes through the coronary arteries whereas in in-vivo setups only a fraction of the total volume travels this route. This could result in differences in attenuation in the myocardium (TAC curve) and therefore the MBF values.

Adequate MBF calculation relies on adequate mixing of iodine contrast agent with the porcine blood. In in-vivo models, the blood and contrast passes through the right side of the heart and the lungs thereby ensuring adequate mixing. To allow adequate mixing in our set-up, the contrast injection was done 200 cm upstream of the aortic root in the tubing of the perfusion loop. The reservoir was placed in a manner that the blood was pumped from the upper part of the blood-reservoir. Recirculation of the contrast medium was minimized by the higher mass of the contrast medium compared the mass of the blood, resulting the contrast agent to accumulate at the bottom of the reservoir. The use of an ex-vivo model allows direct control of the input blood flow, where this is not achievable in in-vivo models. This direct control of inflow and the direct flow through the coronaries gives the unique possibility to compare the calculated MBF values with the true MBF values. These differences in experimental set up could explain the lower values of MBF in shuttle mode in comparison with the study by Bamberg et al. [13]. Bamberg et al. reported mean MBF values of 113 ± 35 mL/100 mL/min corresponding to 1.18 mL/g/min compared to a median of 0.87 mL/g/min in our results.

The non-shuttle and continuous scan protocols were designed with the purpose to increase the temporal sampling rate, specifically for this proof of principle study. These protocols are not meant for clinical purposes, in view of the radiation exposure. In general, the radiation dose will increase with increasing temporal sampling rates. Further studies should investigate what the ideal trade-off is between more accurate MBF estimates and increased radiation dose. For this purpose the protocols should be optimized to reduce radiation while maintaining the image quality. We want to emphazise that the main goal of this study was to investigate the role of temporal sampling rates on quantification of MBF and not to develop a clinical protocol. For optimal clinical use the main goal should not be to get comparable absolute results as MRI by adjusting temporal sampling rates but to reach optimal diagnostic accuracy for all different CT acquisition protocols. It is important to realize that differences in temporal sampling rates highly influence the absolute MBF value and thereby also should be taken into account while using pre-specified thresholds. This becomes more relevant when DSCT system and MDCT systems are compared, since MDCT systems do not use a shuttle mode and operate at different temporal sampling rates.

In conclusion, our results provide experimental prove that limited temporal sampling rates in standard dynamic CTMPI techniques contributes to substantial underestimation of true MBF values. Dynamic CTMPI using increased temporal sampling rates, results in 44–98% higher and more accurate MBF values compared to the currently used shuttle technique.

## Perspectives

### Competency in medical knowledge

Dynamic CT perfusion is a CT technique that offers the possibility to quantify myocardial blood flow and detect perfusion defects. This experimental study showed that the absolute MBF are substantially underestimated as a result of low temporal sampling rates in conventional scan modes. An increase in temporal sampling rates not only increased the absolute MBF values, but also increased the accuracy of the measurement compared to the true myocardial blood flow.

### Translational outlook

Future studies are needed to establish the optimal trade-off between increased radiation dose and more accurate myocardial blood flow estimates. As a consequence of the effect of temporal sampling rates, threshold to determine perfusion defects, should be corrected for the specific temporal sampling rates used.

## Abbreviations

AIF: Arterial input function
CAD: Coronary artery disease
CT: Computed tomography
CTA: Computed tomography angiography
CTMPI: CT myocardial perfusion imaging
Cx: Circumflex artery
ECG: Electrocardiography
FFR: Fractional flow reserve
HU: Hounsfield unit
IQR: Interquartile range
MBF: Myocardial blood flow
MPI: Myocardial perfusion imaging
TAC: Tissue attenuation curve
VPCT: Volume perfusion CT
